# Renal Diseases Associated with Hematologic Malignancies and Thymoma in the Absence of Renal Monoclonal Immunoglobulin Deposits

**DOI:** 10.3390/diagnostics11040710

**Published:** 2021-04-15

**Authors:** Antoine Morel, Marie-Sophie Meuleman, Anissa Moktefi, Vincent Audard

**Affiliations:** 1 Assistance Publique des Hôpitaux de Paris (AP-HP), Hôpitaux Universitaires Henri Mondor, Service de Néphrologie et Transplantation, Centre de Référence Maladie Rare “Syndrome Néphrotique Idiopathique”, Fédération Hospitalo-Universitaire “Innovative Therapy for Immune Disorders”, 94000 Créteil, France; antoine.morel@aphp.fr (A.M.); marie.meuleman@aphp.fr (M.-S.M.); 2 Institut National de la Santé et de la Recherche Médicale (INSERM) U955, Institut Mondor de Recherche Biomédicale (IMRB), Université Paris Est Créteil, Equipe 21, 94000 Créteil, France; anissa.moktefi@aphp.fr; 3 Département de Pathologie, AP-HP, Hôpitaux Universitaires Henri Mondor, 94000 Créteil, France

**Keywords:** hematologic malignancies, glomerulonephritis, acute kidney injury, onconephrology

## Abstract

In addition to kidney diseases characterized by the precipitation and deposition of overproduced monoclonal immunoglobulin and kidney damage due to chemotherapy agents, a broad spectrum of renal lesions may be found in patients with hematologic malignancies. Glomerular diseases, in the form of paraneoplastic glomerulopathies and acute kidney injury with various degrees of proteinuria due to specific lymphomatous interstitial and/or glomerular infiltration, are two major renal complications observed in the lymphoid disorder setting. However, other hematologic neoplasms, including chronic lymphocytic leukemia, thymoma, myeloproliferative disorders, Castleman disease and hemophagocytic syndrome, have also been associated with the development of kidney lesions. These renal disorders require prompt recognition by the clinician, due to the need to implement specific treatment, depending on the chemotherapy regimen, to decrease the risk of subsequent chronic kidney disease. In the context of renal disease related to hematologic malignancies, renal biopsy remains crucial for accurate pathological diagnosis, with the aim of optimizing medical care for these patients. In this review, we provide an update on the epidemiology, clinical presentation, pathophysiological processes and diagnostic strategy for kidney diseases associated with hematologic malignancies outside the spectrum of monoclonal gammopathy of renal significance.

## 1. Introduction

Onconephrology, covering a broad spectrum of renal manifestations occurring in patients with solid neoplasms or hematologic malignancies, has recently emerged as a subspecialty requiring a multidisciplinary approach and continual interactions between nephrologists and oncologists [1]. In this setting, hematologic malignancies are of particular interest, because almost all renal disorders (e.g., acute renal injury, electrolyte disorders, glomerular involvement) may be observed in patients with lymphoid/myeloid disorders or with plasma cell dyscrasia [2,3,4]. In addition to the specific and well-known role of monoclonal immunoglobulin deposition as a key factor in kidney lesions in patients with monoclonal gammopathy of renal significance (MGRS) [4] and the recent demonstration of renal toxicity due to the growing use of immune checkpoint inhibitors [5] or anticancer therapies targeting vascular endothelial growth factor (VEGF) [6], clinicians need to be able to recognize other kidney parenchymal lesions occurring in patients with hematologic malignancies, because these lesions may affect prognosis and interfere with optimal management. This review aims to provide an overview of current knowledge about renal manifestations (unrelated to monoclonal immunoglobulin spike or the renal side effects of chemotherapy) in patients with hematologic malignancies, by focusing on several renal disorders that remain a rare cause of renal injury in these patients. Paraneoplastic glomerulopathies consist of glomerular involvement induced by the production of hormones, cytokines, growth factors and tumor antigens by tumor cells [7]. In this field, the most relevant association is the occurrence of membranous nephropathy (MN) in patients with solid carcinomas [8], but close relationships between minimal change disease (MCD) and Hodgkin lymphoma, non-Hodgkin lymphoma (NHL) and thymoma have also been described [3,8]. Furthermore, some studies have reported a close link between Castleman disease (CD) and thrombotic microangiopathy (TMA) [9,10], myeloproliferative neoplasms and focal and segmental glomerulosclerosis (FSGS) [11,12], hemophagocytic syndrome and collapsing glomerulopathy [13]. All these intriguing associations may shed light on the pathogenesis of the underlying glomerular disorders (Table 1). In addition to paraneoplastic glomerular diseases, the diagnosis of specific tumoral infiltrations into the interstitial and/or glomerular area remains challenging for nephrologists, but such lymphoma infiltrations must be pathologically confirmed rapidly, to prevent delays in specific therapeutic management to reduce the risk of subsequent chronic kidney disease (CKD).

## 2. Epidemiology

**Figure 1:**
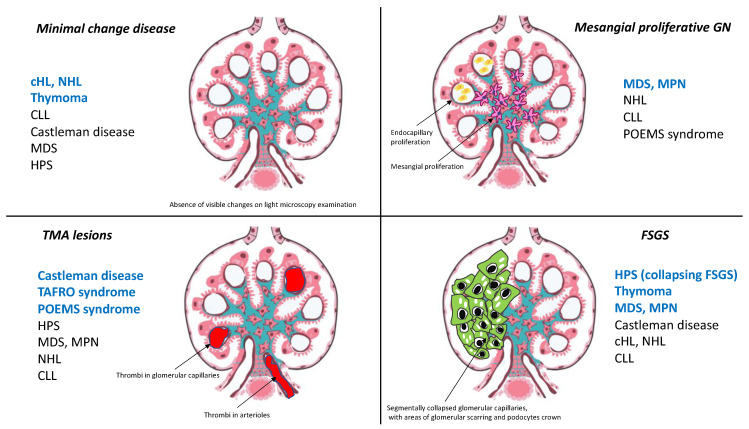
Main glomerular disorders (MCD, TMA, mesangial proliferative GN and FSGS) occurring in association with hematological malignancies and thymoma. The most relevant associations are highlighted in blue in the Figure. cHL: Classical Hodgkin lymphoma; CLL: Chronic lymphocytic leukemia; FSGS: Focal and segmental glomerulosclerosis; HPS: Hemophagocytic syndrome; MDS: Myelodysplastic syndrome; MPN: Myeloproliferative neoplasms; NHL: Non-Hodgkin lymphoma; POEMS: Polyneuropathy, Organomegaly, Endocrinopathy, Monoclonal protein, Skin changes syndrome; TAFRO: Thrombopenia, Anasarca, Fever, Reticulin myelofibrosis, and Organomegaly syndrome.

Every year in the United States, according to the American Cancer Society, about 1,700,000 new cancer cases are diagnosed, 10% of which correspond to hematologic malignancies [14]. Hodgkin (HL) and non-Hodgkin (NHL) lymphomas together account for about 80,000 cases, whereas chronic lymphoid leukemia (CLL) affects 15,000 patients [14,15]. Hematologic malignancies have long been linked to kidney disease, and the list of associations is continually expanding, but the prevalence of kidney disease in patients with hematologic malignancies has not been accurately determined. A prevalence of 7 to 34% has been reported for renal involvement, but these rates vary considerably with the diagnostic method and threshold for proteinuria used [16,17]. Da’as et al. found various renal manifestations directly or indirectly related to lymphoma proliferation in 83 of 700 (12%) patients with NHL and CLL [18]. Li et al. showed that a broad spectrum of biopsy-proven glomerular diseases, associated with or unrelated to specific lymphomatous infiltration, was present in 18 patients with various types of NHL [19]. Similar results, showing diverse glomerular disorders not always directly related to monoclonal immunoglobulin deposition in patients with B-cell lymphoma, were reported by Poitou-Verkinder et al. [20]. The rates and patterns of the associated glomerular lesions vary considerably between underlying hematologic disorders, but some associations seem to be significant, suggesting a close molecular relationship rather than a fortuitous association between the two conditions [3,8]. Strikingly, in both Hodgkin lymphoma (HL) and thymoma, the most frequent associated glomerular disease is MCD [3,21], whereas membranoproliferative glomerulonephritis (MPGN) and MN are the commonest glomerular diseases observed in patients with CLL (36% and 19% of cases of glomerulonephritis, respectively) [22]. Malignant kidney infiltration, classically characterized by bilateral kidney enlargement and acute kidney injury (AKI), is another aspect of renal involvement that can be observed in patients with lymphoproliferative disorders [23]. In a post mortem pathology study of 120 cases with various myeloproliferative and lymphoproliferative disorders, Xiao et al. observed renal involvement in 34% of cases, with a reactive lymphocytic infiltration in 15% [24]. Renal leukemic cell infiltration has been formerly reported in 60% to 90% of cases in autopsy studies of patients with CCL and unimpaired renal function [22]. However, such infiltration remains a common finding in CCL patients (67% of cases) with biopsy-proven renal involvement (AKI or nephrotic syndrome) [20]. Regardless of the underlying B-cell lymphoproliferative disorder, malignant infiltration may be observed as the principal pathological lesion in the kidney and may be associated with some degree of glomerular damage [25]. Hematologic malignancies caused by the clonal proliferation of myeloid cells, or “myeloproliferative neoplasms” (MPNs), include chronic myelogenous leukemia (CML), polycythemia vera (PV), essential thrombocythemia (ET) and primary myelofibrosis (PMF). These conditions are less frequently associated with specific renal involvement, but some glomerular damage has been reported [11,12,26]. Secondary hemophagocytic syndrome (HPS) may occur in various hematologic malignancies, with potential effects on all kidney structures, but via different mechanisms. In these patients, acute tubular necrosis (ATN) is the most relevant renal manifestation, but a renal biopsy should be discussed in cases of suspected HPS-associated glomerulopathy [13,27]. Renal complications seem to be frequent (54%) [28,29] in patients with CD, in whom small-vessel involvement characterized by endotheliosis seems to be the most prominent lesion [9]. The main glomerular lesions occurring in association with hematological malignancies are summarized in Figure 1.

## 3. Hodgkin Lymphoma and Glomerular Disease: The Singular Case of Minimal Change Disease

**Figure 2:**
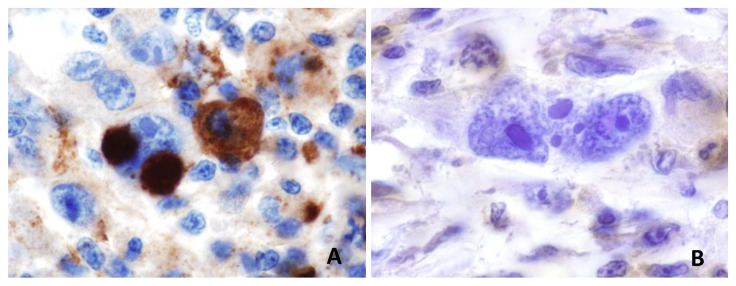
C-mip is selectively induced in HRS cells from patients with MCD occurring in association with cHL (**A**), whereas no expression is found on lymph node tissues from patients with cHL not associated with MCD (**B**).

Classical Hodgkin lymphoma (cHL) is a hematologic disorder characterized by the uncontrolled proliferation of malignant cells known as “Hodgkin and Reed–Sternberg (HRS)” cells. These cells, which are derived from germinal center or post-germinal center B cells, have lost their B-cell phenotype and instead present a highly unusual pattern of expression of many markers from other hematopoietic cell lineages [30]. Many types of glomerulonephritis are recognized as associated with cHL, but the incidence of nephrotic syndrome is low and estimated at about 0.5 to 1% [3]. In two previous studies of 1700 patients with Hodgkin disease, two glomerulopathies were significantly associated with cHL: amyloidosis (0.1% of cases) and MCD (0.4% of cases) [31,32]. The association between cHL and MCD is probably one of the best known among hematologic disorder-related paraneoplastic glomerulopathies [33]. AA amyloidosis has decreased in frequency with current therapeutic strategies, but other glomerular lesions, including FSGS, MN, MPGN and anti-glomerular basement membrane (anti-GBM) or pauci-immune crescentic glomerulonephritis lesions, may also be observed in patients with cHL [2,33,34]. Audard et al. reported the main clinical, biological, pathological and therapeutic characteristics of 21 patients (14 men, mean age of 28.5 years at the time of renal biopsy) with MCD occurring in a context of cHL [34]. Before this study, in the largest reported meta-analysis to date, Eagen et al. reported the simultaneous occurrence of cHL and MCD in 12 of 26 patients [33]. By contrast, Audard et al. reported a median time of 15 months between the diagnosis of MCD and that of cHL (range: 0–156 months) [34]. Nevertheless, in patients suffering from cHL and MCD, the two diseases often appear to follow a remarkably similar course. Indeed, MCD remission occurs after the successful treatment of cHL, regardless of the therapeutic strategy used, suggesting that this glomerular disease is a paraneoplastic disorder when it occurs in the context of cHL [34]. Consistent with this hypothesis, in five patients with steroid- and/or cyclosporine-resistant MCD, the diagnosis and effective treatment of cHL were associated with a complete remission of nephrotic syndrome [34]. These findings were confirmed by Aggarwal et al., who demonstrated that serum permeability activity can be normalized by the use of high-dose chemotherapy to treat cHL [35]. No particular subgroup of patients with cHL, in terms of age, sex, or disease stage, seems to be at a greater risk of developing MCD. Strikingly, in the study of Audard et al. 71% of patients with MCD occurring in association with cHL developed systemic symptoms while 90% of them developed inflammatory syndrome [34]. These findings seem to be quite higher than in patients with cHL without MCD. It has been suggested that MCD is more frequently associated with cHL of a mixed-cellularity histological subtype [36]. However, Audard et al. found that the nodular sclerosing subtype predominated (71.4%) [34]. The pathogenesis of this association remains poorly understood, and the underlying molecular mechanisms are unknown. There is compelling evidence, including clinical and experimental observations, to suggest that both the immune system and podocyte dysfunction are involved in MCD pathogenesis [37]. Several hypotheses have been put forward to explain this intriguing association. Cytokines, such as interleukin-13 (IL-13), secreted by the HRS cells, play an important role in cHL pathogenesis, by stimulating the growth of HRS cells through both autocrine and paracrine effects [38,39]. Interestingly, the IL-13 receptor is expressed in glomeruli from MCD patients, and IL-13 stimulates transcellular ion transport in cultured podocytes [40]. IL-13 does not affect cellular permeability to macromolecules in vitro [40], but transgenic rats overexpressing IL-13 develop proteinuria and MCD-like lesions [41]. Moreover, Nakayama et al. found that tumor necrosis factor-α (TNF-α), was overproduced in patients with relapsing cHL-related MCD, whereas proteinuria decreased simultaneously with TNF-α level normalization after effective chemotherapy [42]. Nevertheless, the role of TNF-α as a potential trigger of proteinuria in patients with primary MCD has not been clearly demonstrated [43]. Audard et al. showed that the c-maf-inducing protein (c-mip) is selectively induced in both podocytes and HRS cells from patients presenting this association, but not in lymphomatous tissue from cHL patients without MCD, suggesting that the overexpression of c-mip in these cells may represent a molecular signature of the association (Figure 2) [44].

## 4. Glomerular Diseases Associated with Non-Hodgkin Lymphoma

Many types of glomerular disease have been described in patients with NHL. In the study by Li et al. including a broad spectrum of lymphoproliferative disorders, renal pathology findings showed MPGN lesions to be the most frequent type of glomerular lesion, occurring in 35% of cases [19]. Crescentic glomerulonephritis was present in 20% and MCD in 17% of cases [19]. Due to the different pathophysiological processes underlying these glomerular lesions and the many subtypes of NHL, no clear direct molecular link between NHL and these glomerular lesions has ever been demonstrated. By focusing on specific cases of MCD occurring in patients with NHL, Kofman et al. identified 18 patients (10 men, mean age at the time of renal biopsy 62.5 years) among 13,992 cases of NHL displaying this association. A marginal B-cell lymphoma or CLL was found in 50% of these patients, whereas Waldenström macroglobulinemia (WM) was found in 33.3% [45].

CLL is characterized by the clonal proliferation of mature lymphocytes with a particular phenotypic signature of CD5-positive B cells circulating within lymphoid organs, such as the spleen, lymph nodes and bone marrow [46]. Several glomerular diseases have been reported in association with CLL [18,22,47,48,49]. Since the first description of nephrotic syndrome in a patient with CLL, the prevalence of nephrotic syndrome has been estimated to be below 1% [50]. Moulin et al. found that MPGN was the most frequent (69%) histological lesion, followed by crescent glomerulonephritis (8%), FSGS (8%) and mesangial hypertrophy (8%) [48]. A recent extensive literature review revealed that MPGN and MN were the most frequent underlying glomerular diseases in 36% and 19% of these patients, respectively [22]. In the study by Strati et al. of 4024 patients with CCL or monoclonal B-cell lymphocytosis, a renal biopsy was performed in 49 patients (1.2%) [47]. This biopsy revealed the presence of MPGN in 10 cases and MCD in five cases. For patients with CCL-related MPGN, glomerular damage frequently seems to be mediated by monoclonal immunoglobulin deposition, particularly in cases of cryoglobulin-related MPGN [20]. MCD observed in these patients may result from a dysfunction of both B and T cells. Nevertheless, leukemic status does not appear to be essential for the development of MCD, which has already been described in monoclonal B lymphocytosis, a pre-CLL state [51]. The molecular mechanisms underlying the pathogenesis of CLL-related MN remain unknown, but one French study reported an absence of anti-PLA2R antibodies in four patients with this association [52].

Renal involvement has been extensively studied in patients with WM, a lymphoproliferative disorder characterized by the presence of a monoclonal IgM protein associated with a lymphoplasmacytic infiltrate of more than 10% on bone marrow biopsy [53,54,55,56,57]. Over a 10-year study period, Higgins et al. analyzed renal pathological findings of 57 patients. They observed non-monoclonal gammopathy-related kidney lesions in 18% of cases (two patients with FSGS, two patients with MCD and one patient with thrombotic microangiopathy (TMA), other renal lesions in five cases) [56]. Similar findings were reported by Vos et al., who found a cumulative incidence of about 5% for WM-related nephropathy (44 cases in 1391 patients studied over a period of 15 years) [57]. The renal lesions consisted of various types of monoclonal IgM-related nephropathy lesions (AL amyloidosis in 25%, other monoclonal IgM-deposition disease/cryoglobulinemia in 23%, cast nephropathy in 9%, light chain deposition disease in 9%), with or without an associated lymphoplasmacytic infiltration (18%). Other types of glomerular lesions appeared to be less frequent (TMA in 7%, MCD in 5% and MN in 2%) [57].

## 5. Lymphomatous Infiltration of Renal Parenchyma

**Figure 3:**
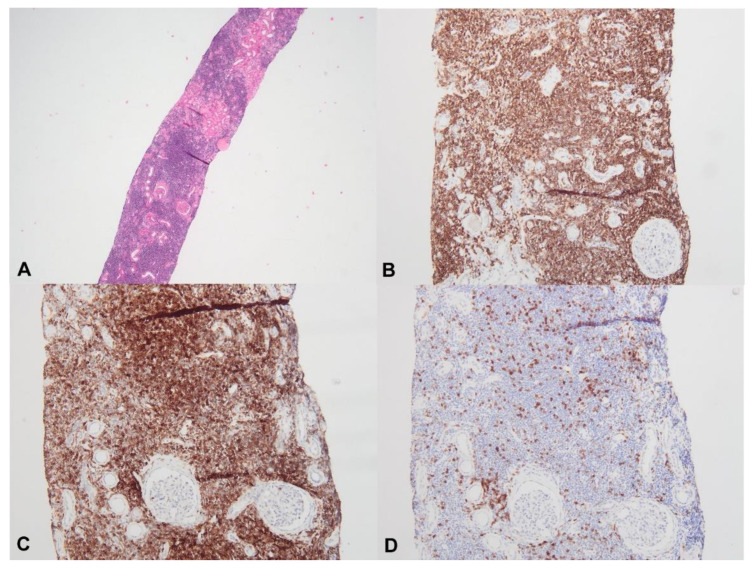
Diffuse interstitial infiltration by chronic lymphoid leukemia cells (**A**), hematein eosin saffron ×20) positive for CD20 ((**B**), ×100) and CD5 ((**C**), ×200), whereas immunohistochemistry with anti-CD3 antibody is negative on tumoral cells (**D**), ×200).

Previous post mortem pathology studies have shown that asymptomatic renal involvement due to lymphomatous parenchymal infiltration by tumoral lymphoid cells is a common finding in patients with advanced lymphoma [22,58,59,60]. Conversely, renal biopsy-proven lymphomatous infiltration appears to be less common in patients with evident renal dysfunction. In a cohort of 700 patients with NHL or CLL, specific tumoral infiltration was found in only five patients with acute renal failure [18]. In their literature review, Uprety et al. found 17 cases of tumoral infiltration in CLL patients, with no clear relationship between absolute lymphocyte count and the risk of developing renal tumoral infiltration (Figure 3) [59].

**Figure 4:**
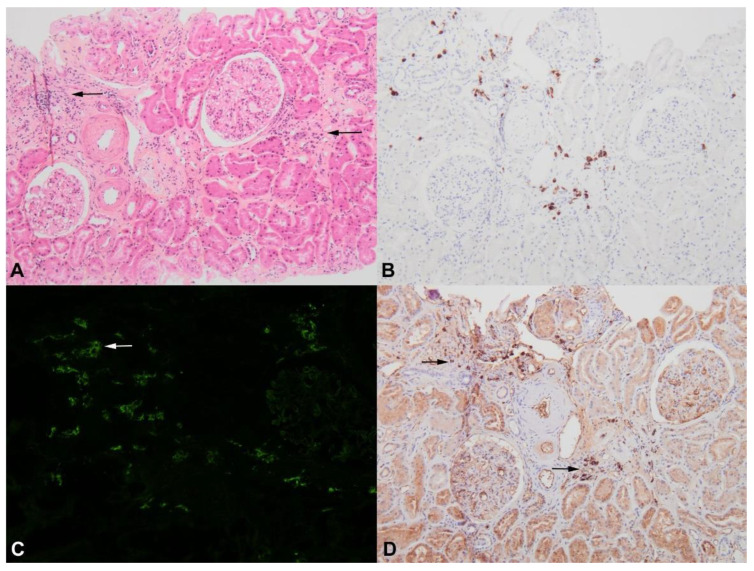
Minimal interstitial infiltration (arrows) by few tumoral lymphocytes and plasma cells ((**A**), hematein eosin saffron ×100), displaying CD79a positivity ((**B**), ×100) and monotypic staining for IgM ((**C**), ×100, arrow) and lambda light chain ((**D**), ×100, arrows).

Lymphoplasmacytic lymphoma infiltration seems to be more frequently recognized in patients with WM (3/14 in the study by Audard et al. [54], 18/35 in the study by Chauvet et al. [55], 6/57 in the study by Higgins et al. [56] and 8/44 in the study by Vos et al. [57]). Törnroth et al. reviewed 55 cases of biopsy-proven renal lymphoma and demonstrated that clinical presentation differed according to the site of the tumoral infiltrate [61]. In this study, AKI was found in 87% of cases in which a predominant involvement of the interstitial area was diagnosed, whereas it was present in 45% of cases in which predominant intraglomerular localization was found. Bilateral kidney enlargement was more evident in patients with interstitial infiltration but was not observed in patients with intraglomerular lymphoma. The definitive pathological diagnosis of malignant cell infiltration may require extensive immunohistochemistry analysis (Figure 4) to distinguish between specific tumoral and interstitial reactive infiltrates, as previously described for patients with cHL or NHL-related MCD [34,45].

**Figure 5:**
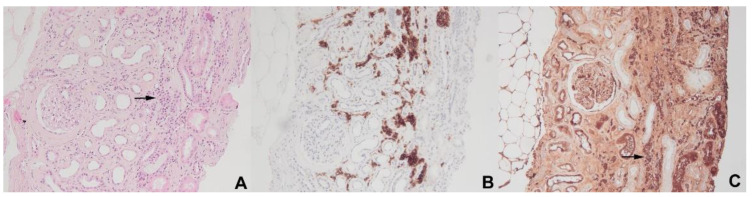
Monotypic plasma cell interstitial nephritis in a patient with multiple myeloma. Numerous interstitial plasma cells ((**A**), hematein eosin saffron ×100, arrow) with strong staining for CD38 ((**B**), ×100) and monotypic staining for kappa light chain ((**C**) ×100, arrow).

Two recent French cohorts including 52 [25] and 34 [60] patients with biopsy-proven malignant B-cell infiltration in the kidney have helped to characterize this renal entity. The hematological diagnosis of a B-cell lymphoproliferative disorder consisting of WM (21/52 and 12/34), CLL (11/52 and 10/34), another low-grade lymphoma (11/52, and 6/34), or diffuse large B-cell lymphoma (8/52 and 6/34) preceded renal manifestations in 29% [25] and 62% [60] of cases. Javaugue et al. reported that AKI and nephrotic syndrome were present at the time of renal biopsy in 56% and 35% of cases, respectively [25]. Corlu et al. noted renal impairment in 85.3% of cases (AKI in 60% of cases) [60]. Consistent with the findings of Törnroth et al. [61], the principal pattern of tumoral infiltration in these two studies was tubulo-interstitial injury (51/52 and in 33/34 cases, respectively) [25,60]. After the initiation of chemotherapy, a renal response was observed in 48% and 27.6% of patients [25,60]. In multivariate analysis, associated immunoglobulin-related nephropathy was found to be the only independent predictor of poor renal outcome [25]. In both studies, tumoral infiltration of the renal parenchyma was associated with poor prognosis (38% and 35.3% of the patients died, after a median follow-up of 21 and 29 months, respectively) [25,60]. Specific renal infiltration by tumoral lymphoid cells seems to be a very rare finding, but has been anecdotally reported during the course of cHL [58] and multiple myeloma (Figure 5) [62].

## 6. Glomerulopathies Related to Myeloproliferative Neoplasms and Myelodysplastic Syndromes

MPN is a group of clonal hematopoietic stem cell disorders characterized by aberrant proliferation of at least one of the precursors of the myeloid, erythroid, granulocytic and megakaryocytic lineages [63]. The main genetic driver mutations associated with these disorders are mutations of the genes encoding Janus kinase 2 (JAK2), or calreticulin (CALR), the Bcr-abl mutation, which is closely associated with CML, and by a truncated form of the thrombopoietin receptor gene [63]. In a cohort of 143 patients with Bcr-Abl-negative MPN, 29% had a CKD stage ≥3 at the time of hematologic diagnosis, and 20% had a rapid annual loss of eGFR (>3 mL/min/1.73m^2^) [64]. Renal manifestations of MPN driven by hyperuricemia [65] and the increase in the risk of renal vessel thrombosis [66,67] in these patients are well known, but specific renal involvement remains a rare finding. The first relevant study regarding glomerular involvement in a context of MPN concerned a cohort of 138 patients, five of whom (3.6%) (two patients with PV, two with ET and one with PMF) had undergone kidney biopsy for the exploration of renal impairment associated with proteinuria of 0.5 to 5.4 g/day [11]. Histological findings led to the identification of FSGS lesions in three cases, associated with diffuse mesangial sclerosis in two cases [11]. In 2011, Said et al. revisited the spectrum of these glomerular diseases and proposed the term of “MPN-related glomerulopathy”, based on a review of the renal biopsy specimens of 11 patients (8 with PMF, and 1 each with CML, PV, and ET) with this association [26]. The authors defined a specific pattern of glomerular damage associated with MPN, characterized by mesangial hypercellularity and sclerosis, which were present in all patients, associated with some degree of segmental sclerosis (73%), features of chronic TMA (82%) and intracapillary hematopoietic stem cell infiltration (36% of cases) without the detection of immune-complex deposits on immunofluorescence or ultrastructural analysis [26]. Interestingly, renal involvement occurred late in the course of MPN (a median of 7.2 years after hematologic disease diagnosis) and all patients had significant proteinuria (median proteinuria of 6.8 g/day). Büttner-Herold et al. recently confirmed these pathological findings by investigating kidney biopsy specimens from 29 patients (23 men, mean age 67 years) with various types of MPN or Myelodysplastic syndrome (MDS) [12]. In this study, the most prominent glomerular lesion included features of both chronic TMA (71%) and acute TMA, with acute endothelial injury (68%), intracapillary platelet aggregation (62%), and mesangiolysis (21%), whereas extramedullary hematopoiesis (EMH) was found in 17% of biopsy specimens [12]. Büttner-Herold et al. found podocytopathy lesions (e.g., foot process effacement >75%) in 8/25 kidney biopsy specimens analyzed by electron microscopy, and 66% of kidney biopsies had features typical of FSGS lesions [12]. However, the existence of a molecular link between these conditions remains to be clarified. High levels of transforming growth factor-β (TGF-β) and VEGF have been found in patients with primary myelofibrosis [68], and these molecules seem to be involved in progression of the hematologic disease [69,70]. Interestingly, TGF-β induces podocyte apoptosis and depletion in both transgenic mice and cultured podocytes, leading to progressive glomerulosclerosis [71]. Belliere et al. described the spectrum of kidney disorders observed in patients with chronic myelomonocytic leukemia (CMML) or Bcr-Abl-negative MPN, by analyzing the various histological patterns in 14 patients: the principal renal lesions consisted of pauci-immune glomerulonephritis (*n* = 5), EMH (*n* = 6), tubular atrophy and interstitial fibrosis with polymorphic inflammation (*n* = 8), IgA nephropathy (*n* = 2) and AA amyloidosis (*n* = 1). Interestingly, immunostaining for CD61 confirmed the occurrence of megakaryocyte infiltration in the glomeruli or interstitium in 5/8 patients, and massive CMML infiltration in the kidney was identified in one case [72]. Another form of renal disease, “renal EMH”, seems to occur in patients with MPN, particularly in those with PMF [73]. In a report of 14 patients (9 men, mean age 68 years) with kidney biopsy-proven EMH, the histological lesions observed consisted of an interstitial infiltrate (*n* = 12) and/or perirenal infiltrate (*n* = 3) and/or mass-like lesions (*n* = 1), but concomitant glomerular lesions were found in all patients. This singular renal entity is characterized by renal impairment in 36% of cases, proteinuria with nephrotic syndrome in 64% of cases, and its definitive diagnosis requires immunohistochemical staining to detect myeloid precursors (myeloperoxidase stain) and megakaryocyte markers (CD61) [73].

MDS is a clonal hematopoietic stem cell disorder characterized by ineffective hematopoiesis, leading to peripheral cytopenia, an increase in the risk of developing autoimmune disease and a tendency toward leukemic transformation in up to 30% of cases [74]. In a cohort of 125 patients with MDS, three patients (2.4%) presented typical features of nephrotic syndrome [75]. A broad spectrum of glomerular diseases, such as mesangial proliferative glomerulonephritis, immunoglobulin-associated MPGN type 1, IgA nephropathy, IgA vasculitis, crescentic C3 glomerulopathy, fibrillary glomerulonephritis, FSGS, MCD, and MN, has been described in association with MDS [75,76,77,78,79]. As the pathophysiological mechanisms underlying these glomerular lesions are different, these findings raise questions about whether there is a clear relationship between the two conditions. In a recent study, Schwotzer et al. retrospectively identified 19 patients with MDS (median age, 74 years, 14 men) who had undergone renal biopsy to explore renal impairment (AKI in 16 cases, median proteinuria 120 mg/mmol) [79]. MDS was diagnosed before renal biopsy in 13 cases and extrarenal manifestations were observed in 58% of cases. Strikingly, the main pathological feature in the kidney was acute tubulointerstitial nephritis, which was present in 37% of cases; immune-related glomerular diseases were found in other patients, suggesting that the kidney may be a target of autoimmunity in MDS patients [79].

## 7. Castleman Disease and POEMS-Related Renal Diseases

**Figure 6:**
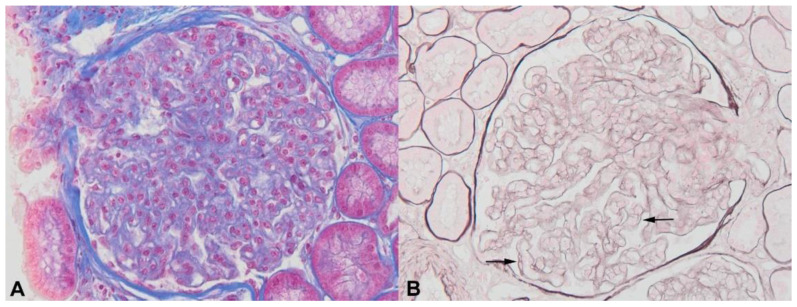
Thrombotic microangiopathy associated with POEMS syndrome. Glomerular enlargement with mesangium loosening, endothelial cell swelling ((**A**), Masson trichrome staining ×400) and double contours ((**B**), Jones methenamine silver, ×400, arrows).

Castleman disease (CD) is a group of lymphoproliferative disorders characterized by angiofollicular lymph node hyperplasia, with two clinical presentations (unicentric/focal and diffuse/multicentric) and three histological variants (hyaline-vascular, plasma-cell and mixed) [80]. Studies of patients with CD have reported prevalence of renal involvement ranging from 25% to 54% [10,28]. El Karoui et al. identified 19 patients (median age, 41.5 years, 10 women) with CD (multicentric in 89% of cases, and with a plasma-cell or mixed variant in 84% of cases) who underwent renal biopsy for the assessment of renal dysfunction and/or significant proteinuria (range: 0.25 to 15 g/day) [9]. In patients without HIV infection (*n* = 15), the most frequent renal histological features were small-vessel lesions (SVLs) with endotheliosis, mesangiolysis, double contours of GBM or arteriolar/glomerular thrombi (in 60% of cases). Other lesions included AA amyloidosis (20%), tubulointerstitial disease (15%), and FSGS lesions in the last patient. The authors found that SVLs were associated with lower levels of VEGF expression on glomeruli. By reviewing 75 published cases of CD (multicentric in 49 cases and unicentric in 26 patients) with biopsy-proven renal involvement, Yuan et al. found that nephrotic syndrome and AKI were present in 28% and 12% of cases, respectively [81]. In this study, the two most frequent underlying renal diseases were amyloidosis and TMA (35% and 17%, respectively). In a third study focusing on 76 patients with CD, renal involvement was found in 25% of cases and was associated with a multicentric clinical subtype and the plasma cell histological variant [10]. Renal biopsy, which was performed in 11 of these 19 patients, demonstrated TMA lesions (55%), crescentic glomerulonephritis (18%), and MCD and chronic tubule-interstitial nephritis in the last two patients. Chemotherapy was associated with renal recovery in 75% of patients with AKI [10]. The spectrum of renal manifestations of thrombocytopenia (T), anasarca (A), fever (F), reticulin (R) myelofibrosis, and organomegaly (O) (TAFRO) syndrome, a severe variant of idiopathic multicentric CD [82], remains poorly described, even though renal dysfunction seems to be a common finding in these patients. An extensive literature review by Nagayama et al. provided a description of kidney biopsy findings for 11 patients (8 women, 3 men, aged from 48 to 84 years) with TAFRO syndrome [83]. All patients had low albumin levels, with various degrees of proteinuria (0.3 to 3.2 g/day), and two main patterns of renal lesions were identified: MPGN-like lesions in five cases and TMA-like lesions in six cases. Furthermore, Mizuno et al. recently reported the clinical and histological characteristics of seven patients with TAFRO syndrome and renal involvement [84]. Clinical features included anasarca (100%), AKI (100%), and low albumin levels (87%), contrasting with a urinary protein excretion rate of <1 g/day in 86% of patients. Glomerular endotheliopathy was a renal pathological hallmark in all cases. The polyneuropathy (P), organomegaly (O), endocrinopathy (E), monoclonal protein (M) and skin changes (S) (POEMS) syndrome is a clinical/pathological entity that may occur on its own or in association with CD [85]. Although not included in the diagnostic criteria, renal damage is commonly observed in patients with POEMS syndrome [28]. AKI often occurs, due to relative hypovolemia resulting from the vascular hyperpermeability that is a hallmark of this disease [86,87]. Nakamoto et al. summarized the main renal manifestations of 52 patients (29 men and 23 women) with POEMS and renal involvement [88]. Proteinuria was absent in 18.4% and below 1 g/day in 77.4%. Renal biopsy specimens, which were available in 22 cases, disclosed two major pathological changes: glomerular alterations characterized by glomerular enlargement, cell proliferation, mesangiolysis, marked swelling of both endothelial and mesangial cells, and endarteritis-like lesions of renal small arteries (Figure 6) [88]. Other rarer renal lesions due to monoclonal immunoglobulin deposition, such as immunotactoid glomerulopathy [89] and light chain deposition disease [90], have also been reported.

## 8. Renal Lesions Occurring in Patients with Hemophagocytic Syndrome

Hemophagocytic syndrome (HPS), also known as macrophagic activation syndrome or hemophagocytic lymphohistiocytosis, is a life-threatening medical condition characterized by multiple organ failure, fever and cytopenia due to the infiltration into the bone marrow and organs of excessive numbers of activated macrophages secondary to inherited disorders, viral, bacterial or fungal infections, autoimmune diseases, solid tumors or lymphoproliferative disorders [91]. AKI due to ATN is frequently observed in HPS patients [13] and is considered to be associated with a poor prognosis [92]. Post mortem studies have revealed that ATN associated with various degrees of interstitial inflammation is observed in up to 45% of patients [13]. In addition to tubular and interstitial injury, glomerular damage may be observed, but is probably underestimated because the poor general condition of some patients may rule out the performance of a renal biopsy. Thaunat et al. performed a retrospective study of a French cohort of nine patients with HPS-associated nephrotic syndrome (together with two additional published cases) [27]. All patients (5 men and 6 women, of African ancestry in 63.6% of cases) had undergone renal biopsy to explore nephrotic syndrome (mean albumin concentration of 17 g/L and a mean proteinuria of 11 g/day). HPS was due to a secondary process in 10/11 patients (T-cell lymphoma (40%), CMV disease (10%), leishmaniasis (10%), B-cell lymphoma (10%) or HL (10%)). NS occurred at the time of HPS diagnosis in 63.6%, or during the course of HPS in 27.4%, (25 ± 30 days after HPS diagnosis). AKI was associated with nephrotic syndrome in 10 patients and required renal replacement therapy in six cases. Death occurred in 64% of cases. The underlying glomerular lesions consisted of MCD in four patients, TMA associated with podocyte injury in two cases and collapsing glomerulopathy (a variant of FSGS) in five cases, but tubulointerstitial damage (interstitial infiltrate, ATN and microcystic tubular dilation) was found in all patients with AKI. The pathophysiological processes involved in HPS-related glomerular disease remain uncertain. In the survey performed by Thaunat et al., 6/11 patients had malignant lymphoid disorders that have been associated with the occurrence of MCD and FSGS (see above). A significant increase in the levels of proinflammatory cytokines (such as TNF-α) was observed in three of the cases studied by Thaunat et al. [27]. Unfortunately, in this cohort, including 63.6% Afro-Caribbean individuals, APOL1 risk alleles, a recently identified major risk factor associated with the occurrence of collapsing glomerulopathy, were not investigated [93,94]. Another glomerular disease has been described as a specific pattern of HPS, defined as “histiocytosis glomerulopathy”. Histological analyses revealed massive and diffuse glomerular CD68^+^ macrophage infiltration associated with intraglomerular hemophagocytosis [95,96].

## 9. Acquired Thymic Disease-Associated Nephropathy

Acquired thymic diseases (thymic hyperplasia or thymoma) are associated with a broad spectrum of autoimmune disorders, including myasthenia gravis, which is found in 15 to 20% of cases, but also autoimmune erythroblastopenia, systemic lupus erythematosus, and thyroid disorders [97,98]. The prevalence of renal involvement seems to be low (<1%) but such involvement is probably underdiagnosed, as the occurrence of renal disease is rarely reported in retrospective surgical series [21,99]. MCD is the commonest paraneoplastic glomerular disease occurring in patients with thymoma, but other glomerular lesions have also been reported [3]. Karras et al. retrospectively investigated the characteristics of 21 patients (8 men, 13 women, mean age 49 years) with kidney biopsy-proven acquired thymic disease-related glomerulopathy [99]. The thymic disease in these patients was mostly malignant thymoma, which was found in 17/19 patients. Renal disease occurred after the diagnosis of thymic disease (108 ± 83 months) in half the patients and 71% of patients presented a paraneoplastic manifestation other than kidney disease. Renal pathology studies identified MCD in 14/21 patients, MN in 4/21, anti-neutrophil cytoplasm antibody (ANCA)-related crescentic glomerulonephritis in 2/21. Thymoma-related MCD and FSGS were steroid-sensitive in 84% of cases, and prognosis was strongly related to thymoma disease outcome (38% of cases died during follow-up). The high prevalence of MCD in the setting of acquired thymic disease supports the hypothesis of a molecular link between these two medical conditions, probably related to a dysregulation of T-cell functions (see above, in the section “Hodgkin lymphoma and glomerular disease: the singular case of MCD”). In this setting, the Buffalo/Mna rat experimental model, which spontaneously develops MCD/FSGS lesions in association with myasthenia gravis-like disease and thymoma, is likely to be particularly useful for elucidating some of the molecular mechanisms involved in this intriguing association [100,101].

## 10. Conclusions

Hematologic malignancy-related kidney manifestations are infrequent, but their prompt recognition by clinicians is essential because appropriate diagnosis appears to be the key to improving overall prognosis. Paraneoplastic glomerulopathies and specific interstitial lymphomatous infiltration are two specific lesions associated with hematologic neoplasms that can be readily recognized through appropriate screening for renal function and proteinuria, leading to a kidney biopsy, which remains the cornerstone of patient management.

## Figures and Tables

**Table 1:** Ig deposits non-related glomerular diseases associated with hematologic malignancies reported in the literature.

Hematological Malignancies	Ig Deposits Non-Related Glomerular Diseases Reported in the Literature
**Thymoma**	MCD, MN, ANCA+ crescentic GN, FSGS, TMA
**Castleman**	TMA, crescentic GN, MCD, FSGS, AA amyloidosis
**TAFRO syndrome**	MPGN-like and TMA-like lesion, glomerular endotheliopathy without thrombi
**POEMS syndrome**	TMA-like lesion, mesangial proliferative GN, endarteritis-like lesion
**HPS**	FSGS, MCD, TMA, Histiocytosis glomerulopathy
**Myelodysplastic syndrome**	Mesangial proliferative GN, FSGS, MCD, IR-like GN, TMA, IgAN, MN, C3 glomerulopathy, fibrillary GN
**MPN disorders**	Mesangial proliferative GN, FSGS, TMA, MPN-related glomerulopathy (specific pattern)
**PV**	FSGS, mesangial proliferative GN, IgAN, TMA, EMH, AML infiltration, IR-like GN
**CML**	MCD, MPGN, MN, IgAN, TMA
**CMML**	IR-like GN, TMA, monocyte (CD61+) infiltration within glomerulus
**PMF**	FSGS, mesangioproliferative GN, EMH, IR-like GN
**ET**	FSGS, mesangioproliferative GN, IgAN, TMA
**Hodgkin lymphoma**	AA amyloidosis, MCD, FSGS, MN, MPGN and anti-GBM or pauci-immune crescentic GN
**Non-Hodgkin lymphoma**	MPGN, crescentic GN, MCD, FSGS, MN, TMA, mesangial proliferative GN
**CLL/SLL/MBL**	MPGN, MN, MCD, FSGS, crescentic GN, mesangial proliferative GN, TMA

TAFRO: Thrombopenia, Anasarca, Fever, Reticulin myelofibrosis, and Organomegaly syndrome; POEMS: Polyneuropathy, Organomegaly, Endocrinopathy, Monoclonal protein, Skin changes syndrome; HPS: Hemophagocytic syndrome; MPN: Myeloproliferative neoplasm; CLL: Chronic lymphocytic leukemia; SLL: Small lymphocytic lymphoma; MBL: Monoclonal B lymphocytosis; CMML: Chronic myelo-monocytic leukemia; MCD: Minimal change disease; MN: Membranous nephropathy; FSGS: Focal segmental glomerulosclerosis; TMA: Thrombotic microangiopathy; GN: Glomerulonephritis; MPGN: Membrano-proliferative glomerulonephritis; IR-like GN: Infection related-like glomerulonephritis; EMH: Extra-medullar hematopoiesis; IgAN: IgA nephropathy.
